# Evolutionary pattern of *Macaca fascicularis* in Southeast Asia inferred using Y-chromosomal gene

**DOI:** 10.1186/s12862-021-01757-1

**Published:** 2021-02-15

**Authors:** Jeffrine J. Rovie-Ryan, Faisal Ali Anwarali Khan, Mohd Tajuddin Abdullah

**Affiliations:** 1 National Wildlife Forensic Laboratory, Ex-Situ Conservation Division, Department of Wildlife and National Parks (DWNP) Peninsular Malaysia, KM 10 Cheras Road, 56100 Kuala Lumpur, Malaysia; 2 Faculty of Resource Science and Technology (FRST), Universiti Malaysia Sarawak (UNIMAS), 94300 Kota Samarahan, Sarawak Malaysia; 3 Institute of Tropical Biodiversity and Sustainable Development (ITBSD), Universiti Malaysia Terengganu (UMT), 21030 Kuala Nerus, Terengganu Malaysia; 4 Fellow Academy of Sciences Malaysia, Level 20, West Wing, Tingkat 20, Menara MATRADE, Jalan Sultan Haji Ahmad Shah, 50480 Kuala Lumpur, Malaysia

**Keywords:** TSPY, SRY, Indochinese, Continental and insular sundaic, Hybridization, Secondary contact

## Abstract

**Background:**

We analyzed a combined segment (2032-bp) of the sex-determining region and the testis-specific protein of the Y-chromosome (Y-DNA) gene to clarify the gene flow and phylogenetic relationships of the long-tailed macaques (*Macaca fascicularis*) in Southeast Asia. Phylogenetic relationships were constructed using the maximum likelihood, Bayesian inference, and the median-joining network from a total of 164 adult male *M. fascicularis* from 62 localities in Malaysia, including sequences from the other regions from previous studies.

**Results:**

Based on Y-DNA, we confirm the presence of two lineages of *M. fascicularis*: the Indochinese and Sundaic lineages. The Indochinese lineage is represented by *M. fascicularis* located northwards of the Surat Thani-Krabi depression region and is introgressed by the *Macaca mulatta* Y-DNA. The Sundaic lineage is free from such hybridization event, thus defined as the original carrier of the *M. fascicularis* Y-DNA. We further revealed that the Sundaic lineage differentiated into two forms: the insular and the continental forms. The insular form, which represents the ancestral form of *M. fascicularis*, consists of two haplotypes: a single homogenous haplotype occupying the island of Borneo, Philippines, and southern Sumatra; and the Javan haplotype*.* The more diverse continental form consists of 17 haplotypes in which a dominant haplotype was shared by individuals from southern Thai Peninsular (south of Surat Thani-Krabi depression), Peninsular Malaysia, and Sumatra. Uniquely, Sumatra contains both the continental and insular Y-DNA which can be explained by a secondary contact hypothesis.

**Conclusions:**

Overall, the findings in this study are important: (1) to help authority particularly in Malaysia on the population management activities including translocation and culling of conflict *M. fascicularis*, (2) to identify the unknown origin of captive *M. fascicularis* used in biomedical research, and; (3) the separation between the continental and insular forms warrants for the treatment as separate management units.

## Background

The most common and abundant *Macaca* species in Malaysia, *Macaca fascicularis* (Raffles, 1821) (also known as long-tailed macaques, crab-eating macaques, and cynomolgus macaques), are widely distributed in nature and occupy vast areas of mainland Southeast Asia (SEA) (Bangladesh, Thailand, Cambodia, Vietnam, Laos, Myanmar, Peninsular Malaysia and Singapore), the Greater and Lesser Sunda Islands (Indonesia, Brunei, and the Malaysian Borneo), and the Philippines, extending across 30° of latitude and 35° of longitude [1]. This species is highly adaptable to disturbed environments and secondary forests, and commonly populate low elevation habitat types favouring seashores and mangrove forests, riverbanks, and swamp forests [2, 3]. In Peninsular Malaysia, they are widespread and populate areas in sympatry with human settlements [4, 5]. At the same time, in the Malaysian Borneo (Sabah and Sarawak), they are distributed throughout the lowlands populating the coastal regions [6].

*Macaca fascicularis* like all the other species within the genus *Macaca* demonstrates a high level of male-mediated dispersal while females are sedentary in nature [7, 8]. This implies that the transfer of genes between groups and populations occurs almost exclusively through the dispersal of males [8]. Moreover, the dominant male exhibits a polygynous mating system, which results in a homogenous gene pool of offspring. Because of this characteristics, particular *M. fascicularis* groups or populations would consist of homogenized Y-chromosomal DNA (Y-DNA) [9].

Y-DNA is uniparentally inherited and as a haploid genetic system, is passed down paternally from male adults to their male progenies. Due to the lack of recombination, Y-DNA has one-fourth of the effective population size (*N*_*e*_) of that of nuclear DNA [9, 10]. Additionally, the polygynous mating behaviour of dominant males further lowers the *N*_*e*_ of Y-DNA loci [9, 11–14]. Nevertheless, the paternal inheritance and the highly dispersal nature of male macaques mean that the information from Y-DNA is important to infer the male-mediated gene flow and to distinguish between contemporary gene flow and ancestral polymorphism [15].

The sex-determining region (SRY) and the testis-specific protein (TSPY) are two unlinked gene segments within the Y-DNA which have been used to infer male-mediated gene flow in primates, particularly in Cercopithecinae [9, 15–23]. Specifically, Tosi, Morales and Melnick [15] and Bunlungsup et al. [23] used both the SRY and TSPY segments coupled with mitochondrial (mtDNA) to investigate the contact zone between *M. fascicularis* and *M. mulatta* in the Indochinese region while Tosi, Morales, and Melnick [9, 16] used a small number of representatives from various region of SEA to investigate the phylogeography of *M. fascicularis*.

The widespread distribution of *M. fascicularis* makes it a prime candidate for the study of phylogeography and evolutionary studies. During the Quaternary Periods (Pleistocene and Holocene), the currently separated landmasses in SEA were intermittently joined and formed Sundaland that permits the dispersal of land mammals, including primates [24–26]. The climatic oscillations, especially during the Pleistocene, including major volcanic eruptions of Mount Toba, would have induced vegetation changes causing the expansions or contractions of habitat that influenced the dispersal of animals [27–29]. Moreover, major river systems, lakes, and mountain ranges may also have acted as a barrier to gene flow [26, 28].

In Peninsular Malaysia, the rising number of cases of human-*Macaca* conflict, particularly involving *M. fascicularis* has prompted the Department of Wildlife and National Parks (DWNP) to implement several population control measures including translocations and culling [5, 30]. Conflict *M. fascicularis* mainly male individuals, have been translocated from conflict urban areas to other locations with a less inhabited human population [5]. Therefore, in this study, by investigating the SRY and TSPY segments, information on whether Y-DNA of *M. fascicularis* consists of continuously related or segregated haplotypes would provide information on whether the translocated individuals posed any risks of contamination to the existing population. Furthermore, taking into account the historical zoogeography and phylogeography of SEA, investigation into the male-mediated gene flow would also provide insight into the hypothesis on the existence of historical or current barrier as well as evidence of recent gene flow among the *M. fascicularis* populations in SEA, particularly in Malaysia.

## Results

### Haplotype mapping, sequence characteristics, and genetic diversity

A total of 654- and 1370-bp of sequence lengths were obtained from the sex-determining region (SRY) and the testis-specific protein (TSPY) segments, respectively, from all 164 male *M. fascicularis* samples. Since both segments are closely linked on the Y-chromosome, the datasets from 287 sequences were combined to produce an alignment of 2032-bp (including indels). All the sequences in this study were registered with the National Center for Biotechnology Information (NCBI) and were given accession numbers, as shown in Additional file 1: Table S1.

**Fig. 1:**
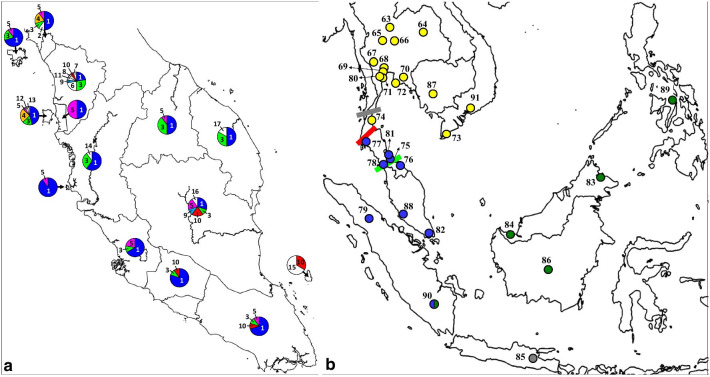
Y-chromosomal haplotypes mapping of *M. fascicularis*. **a** Haplotypes mapping (Haplotype 1–17) used in this study according to the states and major islands of Peninsular Malaysia. Sarawak and Sabah populations formed a single haplotype (Haplotype 18). The numbers correspond to the haplotype designation as listed in Additional file 1: Table S1. Note that only shared haplotypes were coloured. **b** Haplotypes mapping of sequences from the GenBank across Southeast Asia. Blue circles represent Haplotype 1, dark green circles represent Haplotype 18, grey circles represent Haplotype 19, and yellow circles represent Haplotype 20. Grey, red, and green lines indicate the approximate location of Isthmus of Kra, Surat Thani-Krabi depression, and Kangar-Pattani line, respectively. The location numbers in the map corresponds to that in Additional file 2: Table S2

Considering all the *M. fascicularis* sequences (N = 232), 20 haplotypes were observed. Out of the 20 haplotypes, the Malaysian *M. fascicularis* were represented by 18 haplotypes in which 16 were unique to Peninsular Malaysia (Additional file 1: Table S1; Fig. 1a). Peninsular Malaysia constitutes 17 haplotypes where Haplotype 1 is the most dominant and can be found in all the states and major islands (except on Tioman Island). Interestingly, Haplotype 1 is also shared with all nine *M. fascicularis* sequences from the southern Thai Peninsular (south of Surat Thani-Krabi depression), four sequences from Sumatra (Fig. 1b), and the introduced population to Mauritius (N = 10). The Indochinese *M. fascicularis* sequences from Vietnam, Cambodia, Thailand (north of Surat Thani-Krabi depression) formed a single haplotype, Haplotype 20 (Additional file 2: Table S2; Fig. 1b) and were uniquely shared with *M. mulatta* from Burma and South China. The Malaysian Borneo sequences (Sarawak, Labuan Island, and Sabah) formed a single haplotype (Haplotype 18) shared with sequences from Kalimantan and the Philippines including two sequences from southern Sumatra while Java was represented by Haplotype 19.

Based on the haplotype mapping, the *M. fascicularis* sequences were assigned into three geographical groups (except for sequences from Mauritius) for further sequence characterization: (1) Indochinese (Haplotype 20); (2) Continental Sundaic consisting of sequences from the Thai peninsula, Peninsular Malaysia, and Sumatra (Haplotype 1–17), and; (3) Insular Sundaic (Haplotype 18–19). Four indels were observed in *M. fascicularis*, distinguishing between the Indochinese and the Sundaic (continental and insular) haplotypes where two events of; (1) three-bp indels (ACA) at nucleotide position (np) 596–598 and (2) one-bp thymine (T) indel at np 974 occurred (Table 1). Furthermore, within the Sundaic lineage, a single transversion mutation (adenine to thymine) at np 159 was observed on the SRY segment which distinguished the insular Sundaic from the continental Sundaic haplotypes.

**Table 1:** The observed haplotypes and variable sites of the SRY (N = 5) and TSPY (N = 16) segments of *M. fascicularis*

Geographical Groups	Hap. No	N	SRY					TSPY															
													1	1	1	1	1	1	1	1	1	2	2
			1	2	5	5	5	6	7	8	9	9	0	1	3	3	4	6	8	9	9	0	0
			5	0	9	9	9	6	4	0	7	7	9	0	0	6	2	5	6	2	5	1	2
			9	5	6	7	8	2	0	2	4	8	2	8	5	5	1	1	1	4	6	0	7
Continental Sundaic	Hap1	93	A	A	–	–	–	T	C	A	T	A	A	A	A	A	A	A	A	T	G	G	G
	Hap2	1			–	–	–									G							
	Hap3	25			–	–	–											G					
	Hap4	8			–	–	–						G										
	Hap5	12			–	–	–														C		
	Hap6	4			–	–	–						G					G					
	Hap7	1			–	–	–						G					G				T	
	Hap8	1			–	–	–									G						T	
	Hap9	2			–	–	–															T	
	Hap10	6			–	–	–												G				
	Hap11	1			–	–	–											G				T	
	Hap12	1			–	–	–	A															
	Hap13	1			–	–	–													C			
	Hap14	1		C	–	–	–						G					G					
	Hap15	2			–	–	–												G			T	C
	Hap16	1			–	–	–		T									G					
	Hap17	1			–	–	–			C													
Insular Sundaic	Hap18	26	***T***		–	–	–																
	Hap19	1	***T***		–	–	–					G											
Indochinese	Hap20	34	T		*A*	*C*	*A*				***–***			C	C		G						

A single nucleotide polymorphism (transversion mutation at np 159) was observed (in bolditalic font) differentiated the insular Sundaic group from the continental Sundaic group while four indels (np 596–598 and 974; in italic font) distinguished the Sundaic group from the Indochinese *M. fascicularis*

Table 2 summarized the sequence characterization, genetic diversity indices, genetic structure, and differentiation among the geographical groups of *M. fascicularis*. The continental Sundaic is the most variable group displaying the highest number of variable sites (VS) and parsimony-informative sites (PIS). Estimation of the Y-DNA nucleotide diversity (π) among the Sundaic lineage revealed that the continental Sundaic haplotypes had a significantly higher π (4.4 × 10^–4^) compared to the insular Sundaic (0.4 × 10^–4^). The genetic diversity for the Indochinese lineage was incalculable since only a single haplotype was observed. The pairwise fixation index (*F*_*ST*_) calculated among the groups revealed that the Indochinese *M. fascicularis* is highly divergent from both the Sundaic forms (*F*_*ST*_ of 0.90 and 0.99 to the continental and insular forms, respectively) while within the Sundaic lineage, the insular and continental forms are moderately differentiated from each other (*F*_*ST*_ of 0.69). The genetic distances among the haplotypes range from 0.05 to 0.35% (data not shown). Among the geographical groups, the genetic distance between continental Sundaic and insular Sundaic is at 0.15%, while the genetic distances between Indochinese with the continental Sundaic and insular Sundaic is at 0.27% and 0.17%, respectively.

**Table 2:** Sequence characterization (as calculated using MEGA7), standard molecular diversity indices (calculated using DnaSP) as well as pairwise *F*_*ST*_ and genetic distances (%) among the geographical groups of *M. fascicularis*

Geographical Groups		N	Sequence characterization			Diversity Indices			Pairwise *F*_*ST*_ (below diagonal) and genetic distances (%; above diagonal)		
			CS	VS	PIS	NHap	*Hd*	π (10^–4^)	1	2	3
1	Indochinese	34	2028	0	0	1	0	0		0.27	0.17
2	Continental Sundaic	171	2014	12	7	17	0.61	4.40	0.90		0.15
3	Insular Sundaic	27	2025	1	0	2	0.07	0.40	0.99	0.69	

CS = conserved sites; VS = variable sites; PIS = parsimony-informative sites; NHap = number of haplotypes; *Hd* = haplotype diversity; π = nucleotide diversity

### Phylogenetic relationships and estimation of divergences

**Fig. 2:**
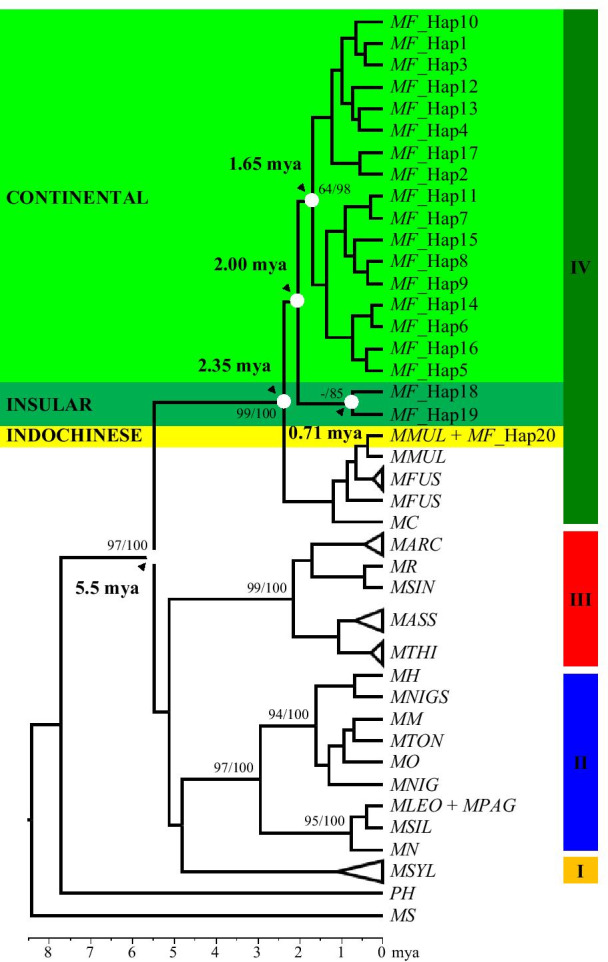
Y-chromosome phylogenetic tree constructed using the haplotypes of *Macaca* as represented by the BI analysis. ML analysis (− lnL = 3809.0846) produced identical topology. Numbers on the branches represents support values for the ML and BI, respectively (only support values above 50% are shown). Roman numerical represents *Macaca* species groupings (I = *sylvanus*; II = *silenus*; III = *sinica*; IV = *fascicularis*). The species nomenclatures are abbreviated as shown in Additional file 2: Table S2

The combined SRY and TSPY phylogenetic relationships constructed by using the maximum likelihood (ML) (− lnL = 3809.0846) and Bayesian inference (BI) methods produced similar topologies and thus were represented by the BI tree (Fig. 2). In general, the tree grouped the *Macaca* into their respective species groupings consistent with the four major lineages of *Macaca*: (1) *sylvanus*-*silenus*, (2) *sinica*, (3) *arctoides*, and (4) *fascicularis*.

**Fig. 3:**
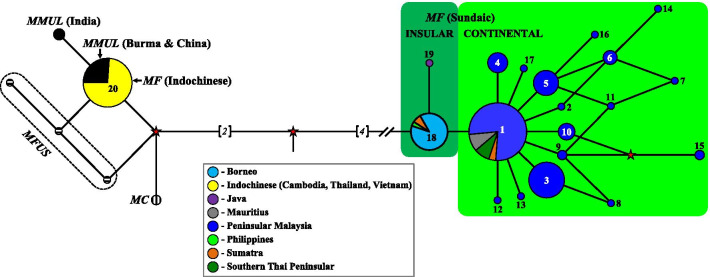
Haplotype median-joining network (MJN) constructed showing the relationships among the species within the *fascicularis* species group. The size of each circle is proportional to the number of individuals in each haplotype. The numbers on the nodes correspond to the haplotype designation as listed in Additional file 1: Table S1, Additional file 2: Table S2. The solid lines connecting the haplotype represent single mutations unless indicated otherwise (italic numbers in parentheses). Hypothetical haplotypes (not sampled) are represented by red stars

Within the *fascicularis* species group, the tree formed two distinct clades: (1) a clade consisting of *M. cyclopis*, *M. fuscata*, *M. mulatta*, and the Indochinese *M. fascicularis*, and (2) *M. fascicularis* haplotypes of Sundaic origin, with high support value (99% and 100% for ML and BI, respectively). Furthermore, the Sundaic *M. fascicularis* lineage further bifurcated into the continental and insular forms but with low support values (below 50%). Similarly, the median-joining network (MJN) (Fig. 3) produced similar groupings, as observed in the phylogenetic tree. Moreover, MJN showed that the continental Sundaic haplotypes were all derived from the insular form, particularly from Haplotype 18. Furthermore, all the other haplotypes within the continental Sundaic haplotypes were derived from the dominant Haplotype 1.

The divergence date estimations calculated by calibrating the BI using the proposed date for the last common ancestor (LCA) of *Macaca* at 5.5 million years ago (mya) are shown in Fig. 2. The *fascicularis* species group (*cyclopis*, *fascicularis*, *fuscata*, and *mulatta*) shared the LCA at around 2.35 mya. Next, the bifurcation between the continental and insular Sundaic forms was estimated to have occurred at ~ 2.00 mya. After that, the continental form shared the LCA at around 1.65 mya while the insular form shared the LCA at around 0.71 mya.

## Discussion

By analyzing 164 male samples from 62 localities throughout Malaysia (including five major islands), this work represents the most comprehensive effort to infer male-mediated gene flow of *M. fascicularis* in Malaysia based on the TSPY and SRY segments of the Y-DNA gene. Combining this dataset with the sequences of *M. fascicularis* from the other regional populations, a complete and broad analysis of the Y-DNA gene flow were able to be estimated, covering the entire range of the species in SEA. In general, the combined segments of the SRY and TSPY in this study were able to provide several paternal insights into the gene flow, phylogenetic relationships at the population level as well as divergence time estimation of *M. fascicularis*.

In this study, the phylogenetic relationship of *Macaca* based on the Y-DNA supported the four major species groupings congruent with the morphological classification by Delson [31]: *sylvanus*, *silenus, sinica*, and *fascicularis* species groups. Although supported with low bootstrap values (except for *fascicularis* species group which is supported by high bootstrap values of 99 and 100% for BI and ML, respectively), all *Macaca* species were clustered into their respective species groups. Similarly, previous nuclear DNA studies also supported the four major groupings [9, 16, 32–34].

Within the *fascicularis* species group, two major clades were observed: (1) a species complex group comprising of *M. cyclopis*, *M. fuscata*, *M. mulatta*, and the Indochinese *M. fascicularis* and (2) *M. fascicularis* haplotypes of Sundaic origin. Both clades shared an LCA at ~ 2.35 mya, which is in proximity to the previous estimation of 2.30 mya by Tosi, Morales, and Melnick [9]. This estimation coincides with the multiple major climate cooling events during Late Pliocene and early Pleistocene occurring between 2.8 and 2.4 mya [35, 36] that influenced the paleoenvironments of SEA [24, 25]. The cold and dry climate during the glacials gave rise to savannah-like vegetation or semi-open woodland [25, 27, 29, 37], causing restriction of dispersal for primates, thus could lead to the separation between both clades.

### Hybridization of *M. mulatta* Y-DNA into *M. fascicularis* in the Indochinese region

The haplotype sharing between *M. mulatta* (N = 12) and *M. fascicularis* (N = 34) represents the male-mediated gene flow of *M. mulatta* into *M. fascicularis* populations from the Indochinese region. Several authors have previously reported on the possible occurrence of the hybridization events between the Indochinese *M. fascicularis* and *M. mulatta* [9, 15, 16, 23, 38–40]. The extent of the contamination of *M. mulatta* Y-DNA into the Indochinese *M. fascicularis* population is not only restricted to the contact zones between both species but possibly more widespread across the Indochinese region [23, 38, 39].

In this study, we confirmed the absence of the male-mediated gene flow of *M. mulatta* into *M. fascicularis* from the Sundaic region, which is restricted at the Surat Thani-Krabi depression region, as shown in Fig. 1b. This is similar to the separation observed between the southern and northern pig-tailed macaques [41]. It is possible that the Surat Thani-Krabi depression played a significant role as a barrier that limits the downwards dispersal of the contaminated Indochinese *M. fascicularis* into the Sundaic lineage. Thus, based on the current finding, we defined the Sundaic *M. fascicularis* as the original carrier of Y-DNA in *M. fascicularis*.

The region of Isthmus of Kra and Kangar-Patani marks the biogeographical barrier that separated the Indochinese and Sundaic zoogeographical regions [42]. These regions display the shift from mixed deciduous forest to the north to wet seasonal rainforest type to the south [43]. Situated along the modern Thailand–Malaysian border (see Fig. 1b), these biogeographical barriers has been documented to limit the gene flow between the Indochinese *M. fascicularis* from their Sundaic conspecifics [15, 23, 39, 44] as well as in other vertebrate faunal species [43, 45–50]. Therefore, to measure the extent of the *M. mulatta* male hybridization into *M. fascicularis*, further research to screen *M. fascicularis* from this area are needed.

### Sundaic *M. fascicularis*: continental and insular forms

The Sundaic *M. fascicularis* lineage, which is the original carrier of Y-DNA in *M. fascicularis*, was further separated into continental and insular forms and shared the LCA at about 2.00 mya. The continental form comprised of haplotypes from southern Thai Peninsular (south of Surat Thani-Krabi depression), Peninsular Malaysia, and Sumatra, including the introduced Mauritian population, while the insular form consisted of haplotypes from southern Sumatra, Java, Borneo, and the Philippines. Likewise, previous molecular studies have identified both forms using mtDNA and nuclear gene [15, 23, 51–56]. Furthermore, the MJN suggested that the insular form was the predecessors of the continental form (Fig. 3). This result would be consistent with the hypothesis that the ancestors of *M. fascicularis* evolved in insular SEA [31, 57, 58]. Wherever and whenever this species originated, the earliest *M. fascicularis*-like fossil was discovered in Java, suggesting that they have already inhibited the Sundaic region as early as 0.9–1.0 mya [58].

After diverging out from the insular form, the continental form diverged into 17 haplotypes, which all shared the LCA at around 1.65 mya (Fig. 2). The dominant Haplotype 1 characterized this form, which is shared with 103 individuals and is distributed as far north as Wat Suwankhuha, of the Thai Peninsular, downwards to entire Peninsular Malaysia until Sumatra of Indonesia. According to the MJN (Fig. 3), all the other continental haplotypes were derived from this dominant haplotype. Moreover, within Peninsular Malaysia, the distribution of haplotypes (Fig. 1a) further showed the segregation of several unique haplotypes that can be found only at the north-western region (Haplotype 4, 6–8, 11–14) as well as the east coast region (Haplotype 15–17). In contrast, the central-southern regions are relatively connected with no unique haplotype defining the region.

Compared to the 17 observed haplotypes within the continental form, only two haplotypes were observed within the insular form, which shared the LCA at around 0.71 mya (Fig. 2). *M. fascicularis* from southern Sumatra, Borneo, and the Sibuyan Island of northern Philippines were all represented by a single dominant insular haplotype, Haplotype 18. The island of Java, on the other hand, was represented by Haplotype 19. The small number of haplotypes observed shows that the insular form consists of significantly low variability and intact Y-DNA. The highly dispersal nature of males would move and homogenize Y-DNA across populations [9] and therefore contribute to this highly intact Y-DNA across the insular region.

Prolong and recurring connectivity that existed in the past could have facilitated the dispersal of males between the insular regions (Sumatra, Java, Borneo, and the Philippines). Several episodes of sea-level fluctuations in Sundaland during the Pliocene up until recently during the Holocene around seven thousand years ago (kya) would permit for such connectivity with some as low as − 120 m below the present level [25, 59]. In addition to the connectivity, a possible ancient bottleneck could explain for the intact Y-DNA in the insular region. The ancestors of the insular form could have been forced into a common refugium postulated to have existed in northern Borneo [24, 25, 29, 60, 61]. Over time, only a single dominant haplotype could have persevered. Alternatively, overhunting and exploitation by early human could also severely have reduced the effective Y-DNA gene pool. Remains of *M. fascicularis*-like species were discovered in cave settlements in Java (~ 0.9 to 1.0 mya) and Borneo (~ 30 to 40 kya) [58] giving evidence to early exploitations by human. Nevertheless, the low number of samples and available sequences (N = 27) could hinder the possibility of detecting other haplotypes within the insular form. Therefore, to further measure the diversity of the insular form, further research utilizing more samples should be conducted on the insular form.

Uniquely, *M. fascicularis* from Sumatra exhibited both the continental and insular Y-DNA haplotypes (Fig. 1b; Additional file 2: Table S2). A secondary contact hypothesis could account for this condition in Sumatra with two possible explanation. The first explanation offered by Tosi and Coke [62] hypothesized that Sumatra was already populated by the insular Sundaic form, prior to the immigrating males of the continental form from the Malay Peninsula. Due to the narrow and shallow waterways on the Sunda Shelf separating the Malay Peninsula and Sumatra [25, 59], a recent dispersal of the dominant continental haplotype from the Malay Peninsula is postulated as being in the process of colonizing the native insular form.

In contrast, a second explanation similar to the first secondary contact hypothesis takes into account the biogeographical history of both regions. Several authors have postulated the formation of several refugia during the Pleistocene, whereas the Malay Peninsula was surrounded by a belt of savannah-like vegetation [24–26, 63]. This implies that dispersal of the continental form occurred from a postulated refugium in Sumatra into the Malay Peninsula and the surrounding areas. Dispersal of the insular form from a refugium in western Java into Sumatra, which was already populated by the continental Sundaic haplotypes could explain for the presence of both forms in Sumatra.

## Conclusions

Findings in this study could also assist the authorities particularly in Malaysia on the population management of conflict *M. fascicularis*. Prior information on the genetic structure is imperative before any measures of population control such as translocation programs or culling are to be conducted [64–66]. Thus, the haplotype mapping in Fig. 1a can be used by the managing authority to correctly manage the local populations without posing risks of contamination to the existing population or accidentally wiping out a unique haplotype due to culling.

Overall, Fig. 1b summarizes the Y-DNA mapping of *M. fascicularis* according to the lineage and forms observed. Evidently, *M. fascicularis* are distributed accordingly to the zoogeographical segregation of SEA. The single nucleotide polymorphism observed at np 159 which distinguishes between the continental and insular forms are important from the perspective of biomedical research. The identification of the genetic background of non-human primate model used in experimental research is important since variations could influence the results and repeatability of experiments [67, 68]. Thus, this finding could assist in the identification of the origin of the captive *M. fascicularis* used in biomedical research, at least paternally.

Finally, findings of this study suggest that the separation between the continental and insular forms warrants for the treatment as separate management units (MUs). MUs are lower ranked conservation units which recognize populations with significant divergence at nuclear or mtDNA and represent populations connected by such low levels of gene flow [65]. The status of the Indochinese lineage, on the other hand, should be handled with caution and until the level of genetic hybridization of Indochinese *M. mulatta* into *M. fascicularis* populations are thoroughly assessed, this lineage should be managed separately from the Sundaic lineage.

## Methods

### Sample collection and GenBank sequences

**Fig. 4:**
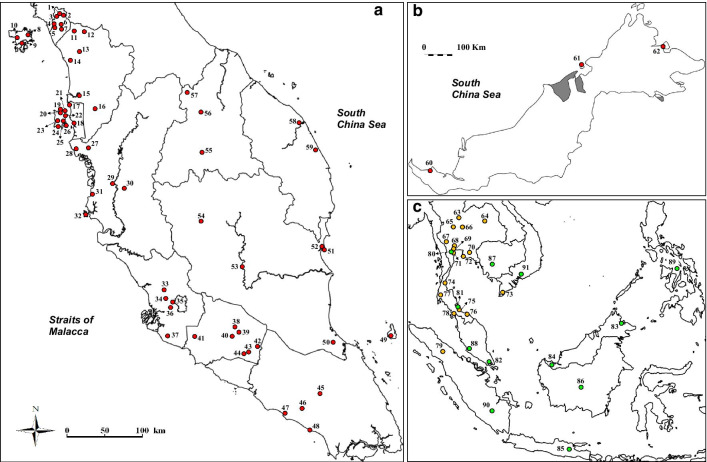
Map showing the localities of the *M. fascicularis* samples and sequences used in this study; **a** from Peninsular Malaysia, **b** from Malaysian Borneo (Sarawak and Sabah), and **c** downloaded sequences from previous studies as obtained from the GenBank (yellow circles represents sequences from Bunlungsup et al. [23] while green circles are sequences from Tosi, Morales and Melnick [9, 15]. The location numbers in the map corresponds to that in Additional file 1: Table S1 (for samples obtained from this study) and Additional file 2: Table S2 (for sequences downloaded from GenBank)

Blood samples were collected from wild free-ranging conflict *M. fascicularis* by authorized personnel and veterinarians of DWNP (for samples from Peninsular Malaysia), Sabah Wildlife Department (for samples from Sabah), and Forest Department of Sarawak (for samples from Sarawak). The euthanization of the conflict animals were also performed by authorized and qualified veterinarians of DWNP. In brief, the macaques were sedated intramuscularly using general anaesthesia (combination of ketamine, 5–10 mg/kg and xylazine, 0.2–0.4 mg/kg) before a lethal dosage of pentobarbital (equal or more than 60 mg/kg) were given intraveneously. A total of 164 samples of adult male long-tailed macaques from 62 localities in Peninsular Malaysia, Sarawak, and Sabah were collected and used in this study (Additional file 1: Table S1). Figure 4a and b summarizes the localities of the samples according to the states and major islands in Malaysia.

Sixty-eight sequences of *M. fascicularis* from other regional populations of were also included in the dataset to make up a total of 232 sequences (Fig. 4c; Additional file 2: Table S2) representing the most comprehensive sampling coverage of the species distribution range. In total, 287 sequences were used for analyses comprising of 20 *Macaca* species and two outgroup species (*Papio hamadryas* and *Mandrillus sphinx*). Both outgroup species were selected as representatives from the major branches within the Family Cercopithecidae.

### DNA extraction, PCR amplification, and DNA sequencing

Total genomic DNA was extracted from blood samples using the QIAamp DNeasy^®^ Blood and Tissue Kit using the protocol provided by the manufacturer (Qiagen, Germany). Two pairs of oligonucleotides were used to amplify the and the TSPY segments of the Y-DNA as designed by Rovie-Ryan et al. [21]. In Old World monkeys the SRY segment is found on the short arm of the Y-DNA [69] while the TSPY segment is typically located on the long arm, close to the centromere region in the Y-DNA [70, 71].

PCR amplifications were conducted in 20 µl reactions using a GeneAmp^®^ PCR System 9700 (Applied Biosystems, USA). Each PCR reaction consists of 1.0 µl of DNA template (~ 15 to 20 ng), PCR mixtures containing 4× Green GoTaq^®^ Flexi Buffer (Promega, USA), 0.875 mM of MgCl_2_ (25 mM), 0.1 mM of each dNTPs (10 mM), 0.1 mM of each primer (10 mM), 1 unit of *Taq* Polymerase (5 unit/µl), and later added with ddH_2_0 to a total of 20 µl of total reaction mixtures. The cycling profile for the amplification was as follows: a preliminary denaturation at 98 °C for 2 min followed by 45 cycles of 95 °C for 30 s, 55 °C for 60 s and 72 °C for 60 s. This was followed by a final extension period of 72 °C for 5 min before the samples were cooled to 10 °C. Cycle sequencing was done on an ABI PRISM^®^377 DNA Sequencer by a sequencing service provider (1st Base Laboratories Sdn. Bhd., Malaysia).

### Sequence characterizations and genetic diversity indices

Multiple sequence alignments were done by using the program Geneious ver5.6 [72]. Prior to further sequence analysis, the SRY and TSPY segments were combined as both segments are closely linked on the Y-chromosome and the partition homogeneity tests conducted in PAUP ver4 [73] did not find significant differences in their evolutionary signal (*P* = 1.0) as also previously reported [9, 16].

Sequence characterizations including conserved sites (CS), VS, and PIS were examined by using MEGA ver6 [74] while DnaSP ver5 [75] were used to calculate the standard genetic diversity indices including the number of haplotypes (NHap), haplotype diversity (*Hd*), and π [76]. Genetic distances were calculated on MEGA ver6 using the Kimura 2-parameter model [77]. To evaluate the amount of population genetic structure pairwise *F*_*ST*_ values were calculated using DnaSP ver5.

### Phylogenetic analysis and divergence time estimates

To infer phylogenetic relationships, the haplotypes data were used to construct phylogenetic trees using the ML, BI and the MJN methods. The best substitution model to run the ML tree and BI was Kimura 2-parameter model with discrete gamma distribution (K2P + G) as determine using MEGA ver6.l. To assess the robustness of the ML tree, bootstrapping [78] with 2000 replicates were conducted. In BEAST package ver2.5 [79], BI was analyzed using two independent runs with 10 million of MCMC chain length each using strict clock settings, Yule model prior [80], and sub-sampled at every 1000 generations. To set the substitution model to K2P + G, HKY85 model [81] was selected with all the base frequencies set to equal and all rate parameters were fixed to 1.0. The convergence of all parameters was assessed using TRACER ver1.6 [82] and both independent runs were then combined using the software LogCombiner ver2.5 available within the BEAST ver2.0 package. A consensus tree was later created from the combined tree files after a burn-in of 10% using the TreeAnnotator ver2.5 also available within the BEAST ver2.0 package. Finally, the MJN was constructed using the Network ver4.6 [83].

To estimate the divergence dates of several important events in the evolutionary and dispersal history of *M. fascicularis* in SEA, the BI tree was recalibrated using the same BI analysis parameters as described above. Using the proposed divergence date of 5.5 mya for the LCA of *Macaca* [9, 31, 84], the priors were set to normal distribution with the mean set to 5.5 and sigma at 0.5.

## Supplementary Information

**Additional file 1: Table S1.** Sample identification, localities, haplotypes designation, and the GenBank Accession Numbers for each of the samples used in this study.

**Additional file 2: Table S2.** Species, sample identification, localities, haplotypes designation, and the GenBank Accession Numbers for downloaded sequences from GenBank used in this study.

## Data Availability

Sequence data produced in this study are available in the NCBI (https://www.ncbi.nlm.nih.gov/) with the Accession No.: KC572634-KC572687; KJ690361-KJ690376; KJ733018-KJ733278.

## Abbreviations

BI: Bayesian inference
CS: Conserved site
DWNP: Department of Wildlife and National Parks
kya: Thousand years ago
LCA: Last common ancestor
MJN: Median-joining network
ML: Maximum likelihood
mtDNA: Mitochondrial DNA
MU: Management unit
mya: Million years ago
NCBI: National Center for Biotechnology Information
PIS: Parsimony-informative site
SEA: Southeast Asia
SRY: Sex-determining region
TSPY: Testis-specific protein
VS: Variable site
